# Knockdown of *Kif20a* inhibits growth of tumors in soft tissue sarcoma *in vitro* and *in vivo*

**DOI:** 10.7150/jca.44777

**Published:** 2020-06-28

**Authors:** Zhenhua Zhu, Zheng Jin, Haibo Zhang, Mei Zhang, Dahui Sun

**Affiliations:** 1 Department of Orthopaedic Trauma, The First Hospital of Jilin University, Xinmin Street 71#, Changchun City, Jilin Province, China.; 2 Department of Immunology, College of Basic Medical Sciences, Jilin University, Xinmin Street 126#, Changchun City, Jilin Province, China.; 3 College of Chemistry, Jilin University, Changchun, Jilin Province, China.

**Keywords:** Kif20*a*, bioinformatics, survival, biomarkers, soft tissue sarcoma

## Abstract

Kif20*a* (Kinesin Family Member 20A), plays a role in cell mitosis, cell migration and intracellular transport. Numerous studies have demonstrated that Kif20a is abnormally highly expressed in a variety of tumors and shows poor prognosis. Soft tissue sarcoma (STS) represents a group of malignant tumors with poor prognosis. The role of Kif20*a* in STSs has not been systematically studied. In the present study, bioinformatics analysis, *in vitro* and *in vivo* experiments were conducted to investigate the function of Kif20*a* in STSs. In bioinformatics analysis higher *KIf20a* expression indicated a poor prognosis. Functional enrichment analysis indicated that *Kif20a* may be related to cell cycle, p53 and other signaling pathways. *In vitro* experiments showed that after the down-regulation of *Kif20a*, cell proliferation, migration and invasion were decreased, while apoptosis was increased. *In vivo* experiments revealed that *Kif20a* affected the proliferation of tumors in tumor-bearing mice. In summary, our findings revealed that *Kif20a* performs an important role in STS, indicating that it is a potential prognostic biomarker and potentially representing a therapeutic target for the disease.

## Introduction

Soft tissue sarcomas are a rare group of malignancies of mesenchymal origin and can occur in various parts of the body, especially the extremities 1. In addition, STSs are highly malignant and prone to lead to lung metastasis 2. At present, surgery, chemotherapy and radiotherapy remain the primary treatment options for STSs 3. However, a number of complications exist, such as postoperative recurrence and metastasis, poor prognosis and poor chemotherapeutic efficacy. As molecular pathology has developed, it has been confirmed that multiple genes are associated with the progression of soft tissue sarcomas. However, no clinical biomarkers have been identified that guide prognosis.

Kinesin-like proteins, such as Kif20*a*, are microtubule-associated motors that mainly play a role in cell mitosis, migration and intracellular transport 4-6. Kif20*a* is abnormally highly expressed in various tumors, such as gastric cancer 7, glioma, pancreatic cancer, breast cancer and bladder cancer 8. Kif20*a* expression has been identified as a prognostic indicator for ovarian clear cell carcinoma 9, nasopharyngeal cancer 10 and pancreatic cancer 11. In breast cancer, Kif20*a* plays a role in the resistance to Taxol 12.

Despite the key role KIF20a plays in numerous tumors, no study has so far explored the relationship between Kif20*a* and STSs. In this study we intended to investigate the expression of Kif20a in STSs and explore its role in carcinogenesis and metastasis. In this study, the expression and function of *Kif20a* in STSs were firstly analyzed by bioinformatics analysis. The function of *Kif20a* was then verified both *in vitro* and *in vivo* by constructing *Kif20a* knockdown cell lines of STS.

## Materials and Methods

### Data sources

The normalized level 3 RNAseq data and relevant clinical characteristics of TCGA-SARC project were downloaded from the UCSC Xena platform (https://xenabrowser.net) 13.

### Survival analysis

Samples from different subtypes were divided into two groups (high vs. low) representing a comparison with the median expression level of *Kif20a*, then survival analysis was performed that compared the two groups.

### Co-expression analysis

A protein-protein interaction (PPI) network of Kif20*a* was constructed using the STRING database (https://string-db.org/) 14, based on both experimentally validated and predicted connections.

### GSEA analysis

Gene set enrichment analysis (GSEA) 15, 16 is also known as functional enrichment analysis, which can determine whether there is statistical difference in the predefined set of genes between two groups of samples. TCGA-SARC data were used to conduct GSEA on samples where *Kif20a* expression level was within either the bottom or top quartile.

### Cell lines and cell culture reagents

Mouse STS cell line WEHI164 was purchased from CCTCC (China Center for Type Culture Collection) (Wuhan, China). Mouse STS cell lines MCA101 and MCA207 and mouse fibrocyte cell line L929 were obtained from Zhu's laboratory (Changchun, China). All the cell lines were maintained in RPIM 1640 (Gibco, USA) supplemented with 10% FBS (Hyclone, USA) and 1% antibiotics (penicillin-streptomycin, Gibco, USA). The culture medium was exchanged every 2-3 days. After culture to 80% confluence, the cells were trypsinized with trypsin-EDTA (0.25%) (Hyclone, USA) for passaging.

### Transfection

A plasmid containing *Kif20a* shRNA was constructed by GIEL (Shanghai, China) using GV102 as the carrier. The sequences of the shRNA and control strands were: 5'- GCCACTCACAAATTTACCTTT-3' and 5'- TTCTCCGAACGTGTCACGT-3', respectively. All the three sarcoma cell lines were seeded into six well plate (Corning, USA) at 5×10^5^ cell/well and cultured overnight. PEI Transfection reagent (Polysciences, UK) was used in accordance with the manufacturer's instructions. After 48h of continuous culture, Geneticin was used to screen the transfected sarcoma cells at concentrations of 400 μg/ml, 600 μg/ml and 600 μg/ml for WEHI164, MCA101 and MCA207, respectively. After expansion culture, RT-qPCR and Western blots were used to confirm that Kif20*a* expression had been silenced.

### Antibodies and Western blotting

The three cell lines (WEHI164, MCA101, MCA207) were collected into EP tubes using a cell scraper, then lysed using RIPA buffer (Beyotime, China) containing protease inhibitor (Complete, Germany). After lysis for 30min on ice, each lysate was centrifuged for 10min at 10,000 g at 4 °C in a microfuge (Eppendorf, Germany). Loading buffer was added and the protein mixtures were heated to 100 °C for 5 minutes for denaturation. Gel electrophoresis was conducted using 12% Bis-Tris prefabricated gel (Genscript, China), an equal volume of protein sample added to each pore. Following separation by electrophoresis, proteins were transferred to PVDF membranes (Sigma, USA) using semi-dry transfer (Bio-Rad, USA) then blocked with blocking buffer (Beyotime, China) for 15min. Membranes were incubated with primary antibodies overnight. After washing with PBST (Genescript, China) 3 times, the corresponding secondary antibody was added and incubated for 1 hour at room temperature. The Western blots were developed using enhanced chemiluminescent reagent (New Cell & Molecular Biotech, China) prior to observation. The primary antibodies used in this study were directed against GAPDH (1:2000, Cell Signaling Technology, USA) and Kif20*a* (1:100, Santa Cruz, USA). The secondary antibodies were goat-anti mouse (1:2000, Proteintech, USA) and goat-anti rabbit (1:2000, Proteintech, USA).

### CCK-8 cell proliferation assay

Cells were counted and seeded into 96-well plates at a density of 5×10^3^ cells/well then cultured in incubator at 37 °C in an atmosphere containing 5% CO_2_ for 12h, 24h, 36h and 48h. Ten μl CCK-8 reagent (Beyotime, China) were then added to each well, then incubated for a further 1h in the incubator (Thermo, USA). Absorbance at a wavelength of 450nm was measured a microplate reader (Bio-Rad, USA).

### Cell cycle analysis

According to the quantity of DNA in each cell in different phases of the cell cycle, the distribution of cells in each phase can be ascertained. The Kif20*a* knockout cells and control cells were seeded in 6-well plates at 3×10^5^ cells/well then 1 ml serum-free culture medium was added to each well to starve the cells for 12h, to ensure that they were at the same phase in the cell cycle. The culture medium was removed and the cells were washed with PBS 3 times and complete culture medium added. After 48 hours, the cells were trypsinized off and washed with PBS, then placed in 70% ice-cold ethanol in a 4℃ refrigerator overnight. After washing with PBS three times, propidium iodide (PI) dye (Beyotime, China) was added and incubated at room temperature for 30 minutes. The cells were placed into flow cytometry tubes after passing through a 200-mesh filter and analyzed by flow cytometry (BD-FACSCalibur, USA), detecting red fluorescence at an excitation wavelength of 488nm. Output data were analyzed using ModFit LT 5.0 (USA, Verity Software House).

### Colony forming assay

The cells were seeded in 6-well plates at a density of 500 cells/well and 1 ml complete medium containing 20% fetal bovine serum was added to each well. When the cell dividing the fastest had formed a colony of at least 50, 1 ml 4% paraformaldehyde was added to each well, fixed at 4℃ for 1h and washed once with PBS. One ml crystal violet was added to each well then stained for 10min. The cells were then washed with double distilled water several times, dried and imaged, then counted using Image J (v 1.52).

### Apoptosis analysis

The cells were seeded in 6-well plates at a density of 1×10^5^/ well. After 48h, cells were digest using trypsin (EDTA free) and collected in EP tubes. After washing with PBS and binding buffer, 100μl binding buffer was added to each tube to resuspend the cells. Then 5 μl Annexin V-PE and 5 μl 7-ADD solution (Sungene biotech, China) was added to each tube. After incubated in dark for 15min, cells were detected in flow cytometry (BD-FACSCalibur, USA).

### Wound healing and transwell assays

Fifty thousand cells were seeded into each well of 6-well plates, and complete medium containing 10% fetal bovine serum added to each well. After 12 hours, a 200 μl pipette tip was then used to generate a scratch in the cell culture. The medium was replaced with complete medium containing 5% fetal bovine serum and the cells were cultured in an incubator at 37 °C. The wound was then photographed with an inverted microscope (Olympus, Japan) at the point of wound creation and after 24h.

To conduct a migration assay, 100 μl Matrigel (Corning, USA) were added to the chamber (8.0 μm pore) membrane, then 1×10^5^ cells in serum-free medium were seeded into the upper well and 500μl RPMI 1640 medium supplemented with 20% FBS added to the lower well. After incubation at 37℃ for 24 hours, the cells and Matrigel in the upper chamber were gently removed with a cotton tip. The membrane was then soaked in 4% paraformaldehyde (Solarbio, China) and fixed at 4 °C for 1h. After soaking then washing in PBS for 1 min, the membrane was soaked in 1% crystal violet solution for 10 min. The membrane was then washed in double distilled water 3 times then dried. The membrane was viewed using an inverted microscope (Olympus, Japan) and multiple fields imaged at random.

### Tumor-bearing mouse model

All animal experiments were performed following the approval of the First Hospital of Jilin University Animal Ethics Committee and in accordance with the Guidelines for the Care and Use of Laboratory Animals of Jilin province. A total of 10 Balb/c or C57 mice per group (6-8 weeks old; weighing 20±2 g) were provided by the Vital River Laboratory (Beijing, China). WEHI164 cells were originally derived from Balb/c mice and MCA101 and MCA207 cells from C57 mice, so both Balb/c and C57 mice were used for the tumor-bearing animal models. Transfected cells were collected and resuspended in 200 μl PBS (Gibco, USA). WEHI164 cells were suspended at a concentration of 1×10^5^ cells/200 μl, and MCA101 and MCA207 cells at 0.5×10^5^ cell/s200 μl. The cells were injected subcutaneously into the lower flank of the mice. After 3 weeks of culture, the animals were sacrificed. Each tumor and main organs were separated, and the length and width of the tumor tissue were measured and weighed. Tumors and organ tissues were fixed with 4% paraformaldehyde, then stained with HE and Ki67 (Proteintech, USA) using immunohistochemical staining.

### Immunohistochemical staining

Fixed tumor tissues were embedded in paraffin and cut into 3 μm slices. Sections were processed for antigen retrieval and stained. Anti-Ki67 (Proteintech, China) was used for immunohistochemical staining (IHC).

### Histological analysis

Paraffin embedded tumor tissue was sectioned to a thickness of 5 μm then stained with hematoxylin and eosin. All samples were examined immediately and imaged using an Olympus (BX53, Japan, ×200).

### Statistical analysis

Bioinformatics analysis was conducted using R programming language (v 3.6.0) and additional software packages. CCK-8 results were analyzed and plotted using GraphPad Prism 8.0.2. The cell cycle results were analyzed by ModFit LT 5.0, and the nucleic acid content in G2 phase was set to be 1.9 times of that in G1 phase. After that, the positions of G1 and G2 peaks were adjusted to make the fitting shape of G1 and G2 phase most consistent with the frequency distribution of nucleic acid content. FlowJ v10.0 software was used for apoptosis analysis to calculate the proportion of quadrants. The clone formation experiment, scratch experiment and Transwell experiment used ImageJ 1.52k for counting statistics. Immunohistochemical staining was assessed using ImageJ 1.52k. After threshold adjustment, the proportion of positive areas was calculated. The difference between groups was calculated using the unpaired t test.

## Results

### High transcription levels of *Kif20a* exhibited poor prognosis in STS

**Figure 1:**
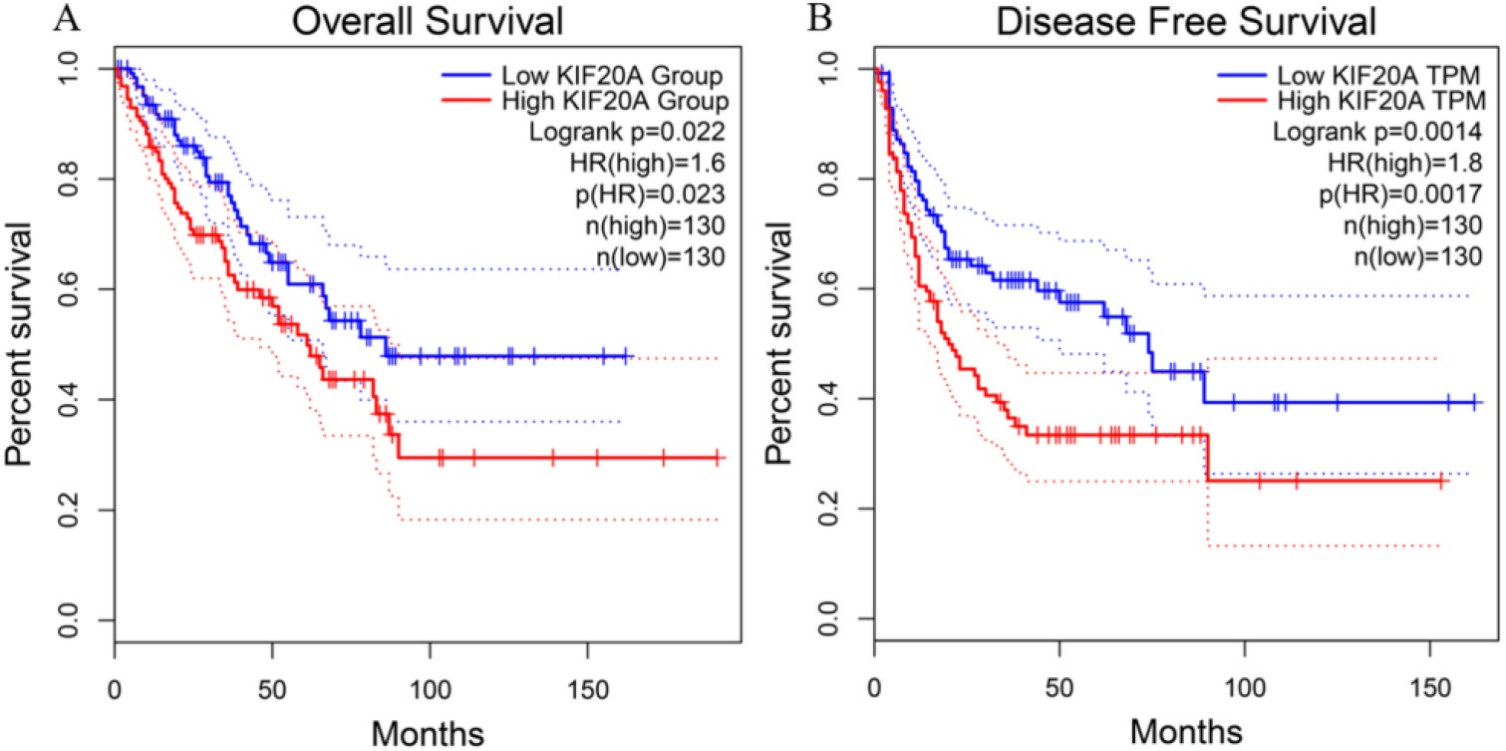
Higher expression of KIF20A resulted in significantly lower rates of (**A**) overall survival and (**B**) disease-free survival.

To investigate the relationship between Kif20a transcription level and STS prognosis, the clinical characteristics and expression data of samples collected in the TCGA-SARC project were analyzed. Patient survival and Kif20*a* expression were analyzed using GEPIA2 (http://gepia2.cancer-pku.cn/) 17. High Kif20*a* expression levels compared with median expression levels in all samples exhibited significantly (*p*<0.05) lower overall survival and disease-free survival rate (Figure 1).

### Kif20*a* predicted protein-protein interaction analysis

**Figure 2:**
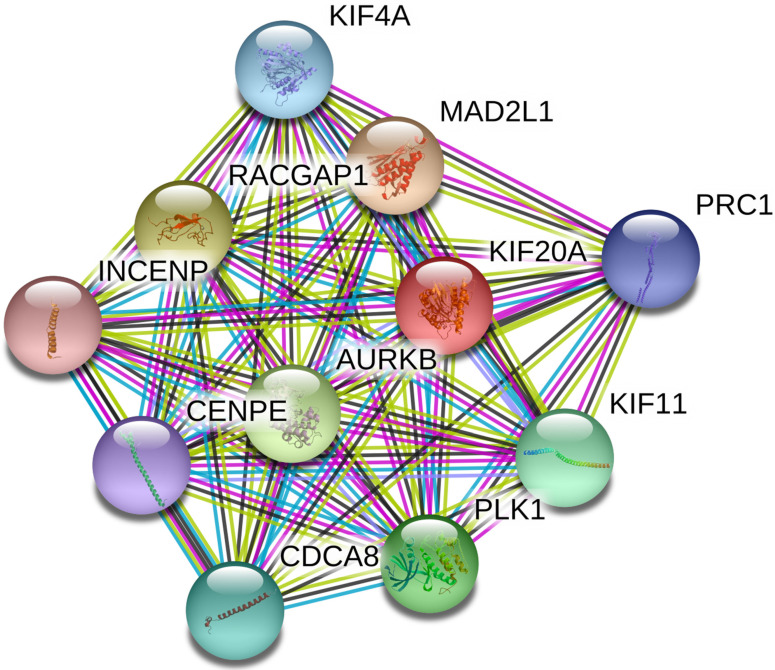
Protein-protein interaction network (PPI) centered on KIF20A.

Based on the data from the STRING database, a PPI internetwork (PPI) including 11 nodes and 55 edges was constructed (PPI enrichment p-value: 9.99e-16) and visualized using Cytoscape software (Figure 2). Co-expression analysis revealed that Kif20*a* was co-expressed with centromere protein E (CENPE), cell division cycle associated 8 (CDCA8), inner centromere protein (INCENP), Rac GTPase-activating protein 1 (RACGAP1), aurora kinase B (AURKB), polo like kinase 1 (PLK1), kinesin family member 11 (KIF11), MAD2 mitotic arrest deficient-like 1 (MAD2L1), kinesin family member 4A (KIF4A) and protein regulator of cytokinesis 1 (PRC1).

### GSEA identified the biological functions and pathways associated with Kif20*a*

**Figure 3:**
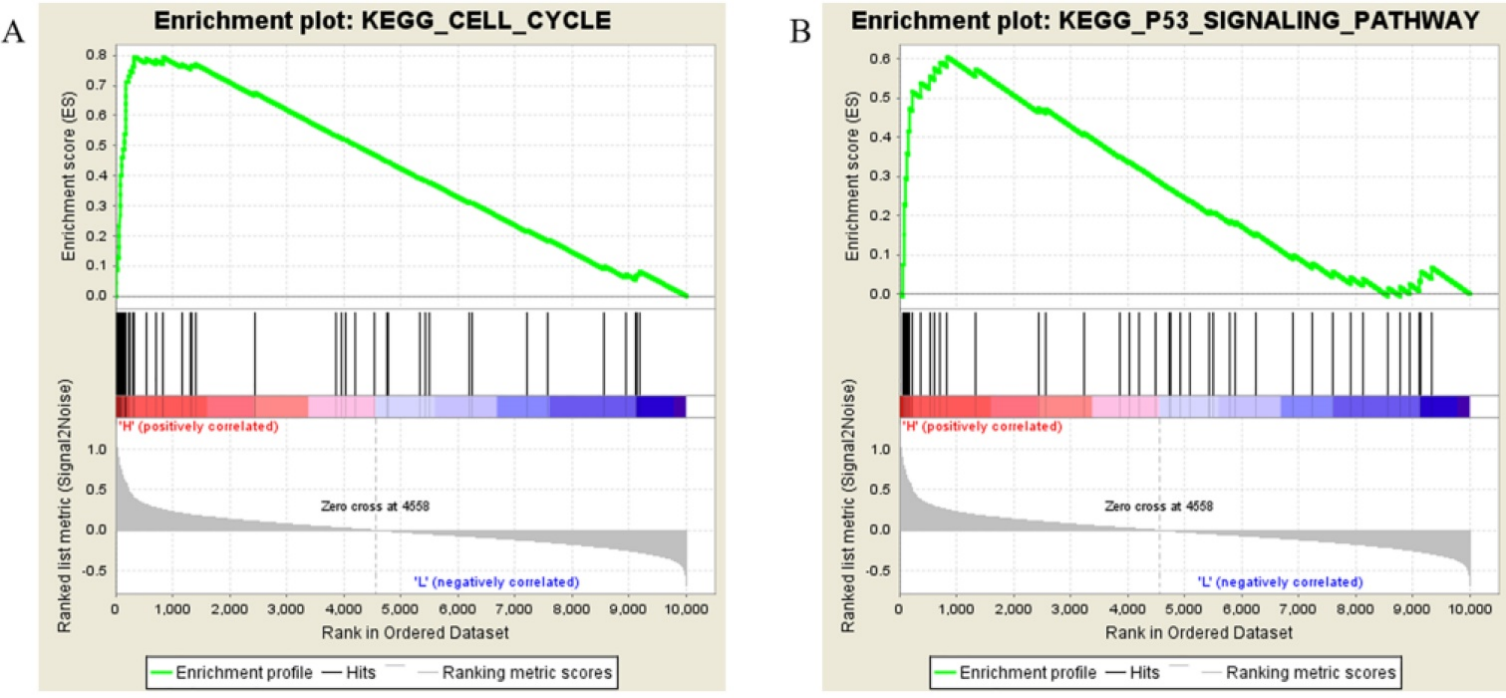
Results of gene set enrichment analysis. Samples with higher expression of KIF20A were significant enriched in (**A**) cell cycle and (**B**) p53 pathways.

Samples from the TCGA-SARC project were divided into two groups (H vs L), the threshold defined by the median Kif20*a* expression level. Followed by gene set enrichment, the high Kif20*a* expression group was enriched in cell cycle function, cell cycle pathway and p53 signaling pathways (Figure 3, Table 2).

### Kif20*a* is upregulated in STS cell lines

**Figure 4:**
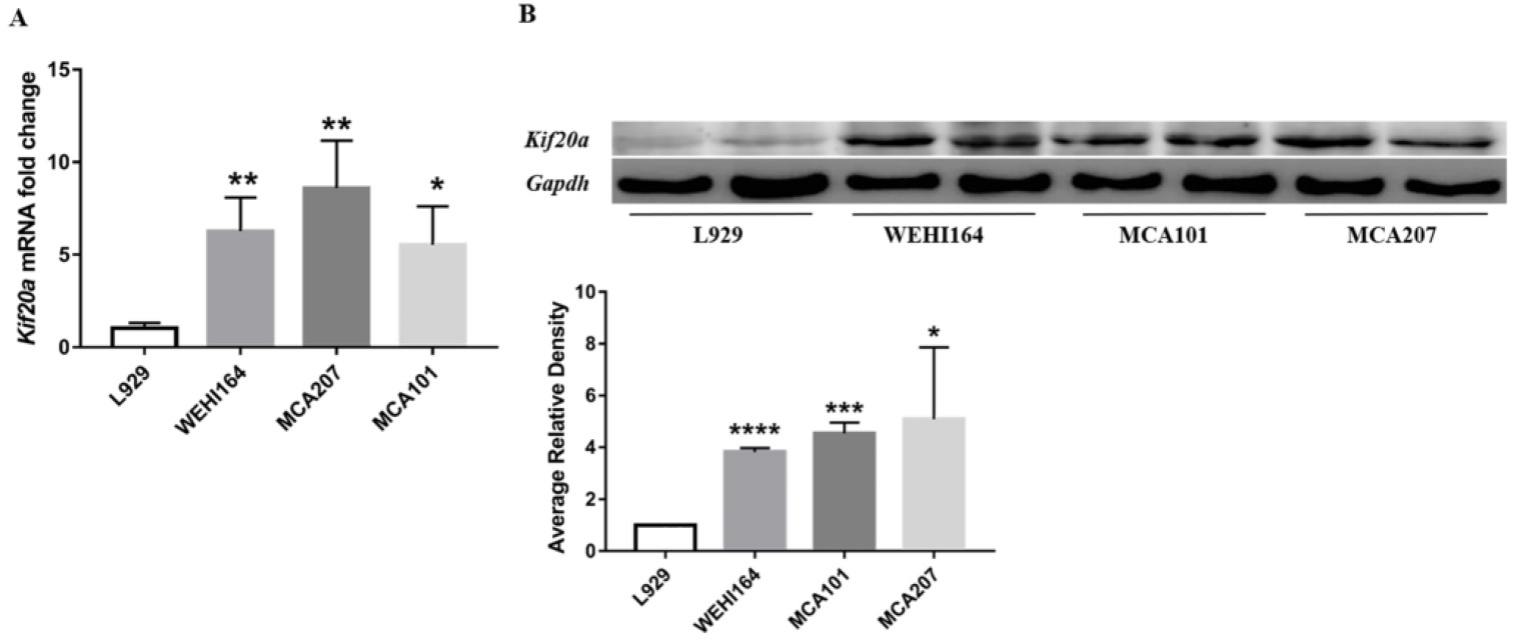
Kif20a was upregulated in STS cell lines. (**A**) Relative Kif20a mRNA expression was determined in three STS cell lines (WEHI164, MCA207, MCA101) and immortalized L929 cells by qRT-PCR and (**B**) by Western blotting. qRT-PCR and Western blot assays were normalized to GAPDH. *P<0.05, **P<0.01, ***P<0.001, ****P<0.0001.

In order to explore Kif20*a* expression in STS, we assessed mRNA and protein expression levels in three STS cell lines (WEHI164, MCA101, MCA207) and L929 cells by RT-qPCR and Western blotting. There was a significant (*p*<0.05) higher Kif20*a* expression level in all STS cell lines (WEHI164, MCA101, MCA207) compared with the L929 control cells (Figure 4).

### Downregulation of *Kif20a* inhibits proliferation *in vitro*

**Figure 5:**
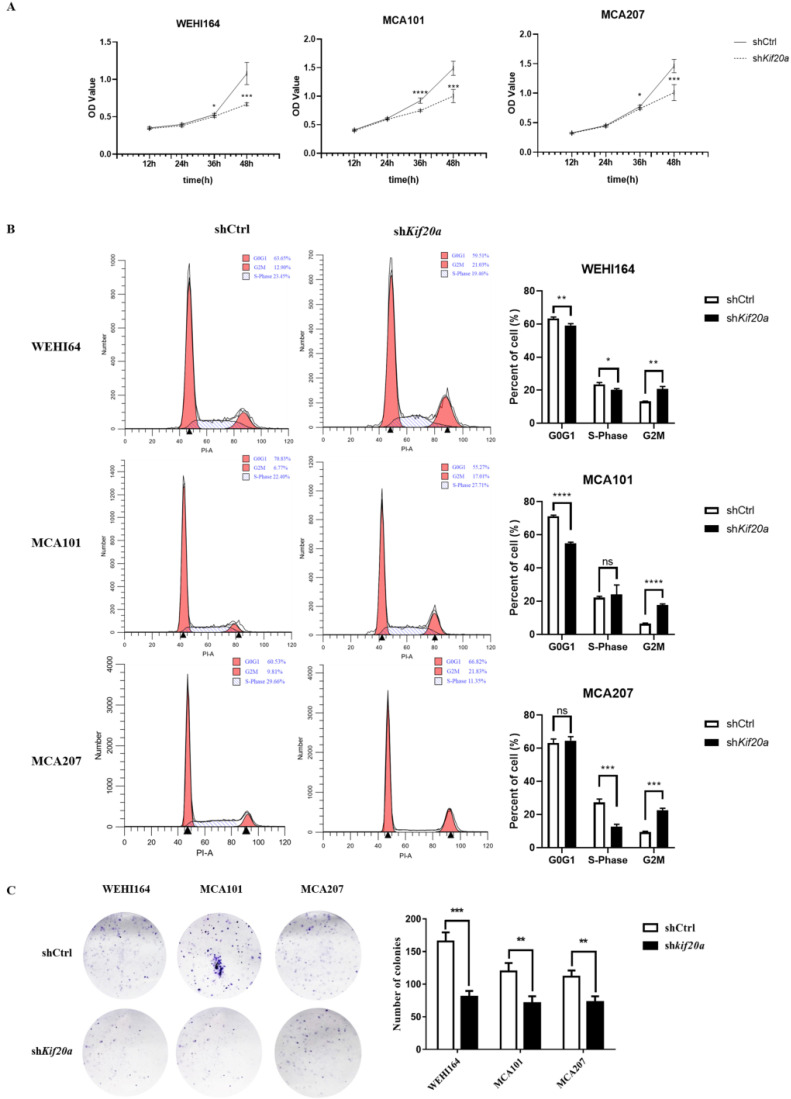
Downregulation of Kif20a inhibited proliferation and colony formation *in vitro*. (**A**) Cell viability was determined by CCK8 assay. (**B**) Cell cycle distribution was determined by flow cytometry. (**C**) Cell colony formation was determined by colony forming assay. ***P<0.001, ****P<0.0001.

To investigate the biological function of Kif20*a*, three STS cell lines (WEHI164, MCA101, MCA207) were transfected with a *Kif20a* shRNA plasmid (shKif20*a* cells) and control plasmid (shCtrl cells). Transfection efficiency was determined by fluorescence microscopy, RT-qPCR and Western blotting analysis. We found that almost all cells were transfected by plasmids (Fig. S1A) and Kif20*a* was successfully knocked down at both mRNA (Fig. S1B) and protein expression levels (Fig. S1C) in WEHI164, MCA101 and MCA207 cell lines. These data indicate that *Kif20a* shRNA plasmids specifically and significantly inhibited *Kif20a* expression in STS cells. To determine the potential effects of *Kif20a* on cell proliferation, CCK-8 analysis was performed. Cell viability was found to decrease in sh*Kif20a* cells (Figure 5A). FACS analysis was used to explore cell cycle in shCtrl and sh*Kif20a* cells. The results demonstrated that G2 phase arrest occurred in all three STS cell lines after Kif20*a* gene knockdown (Figure 5B). A clonogenic assay further confirmed that in Kif20*a* knockout cells, colony formation was also significantly (*P*<0.05) inhibited in the STS cell lines (Figure 5C). These results suggest that downregulation of Kif20*a* inhibited cell proliferation in STS cells by regulating cell cycle distribution.

### Downregulation of *Kif20a* promote cell apoptosis *in vitro*

**Figure 6:**
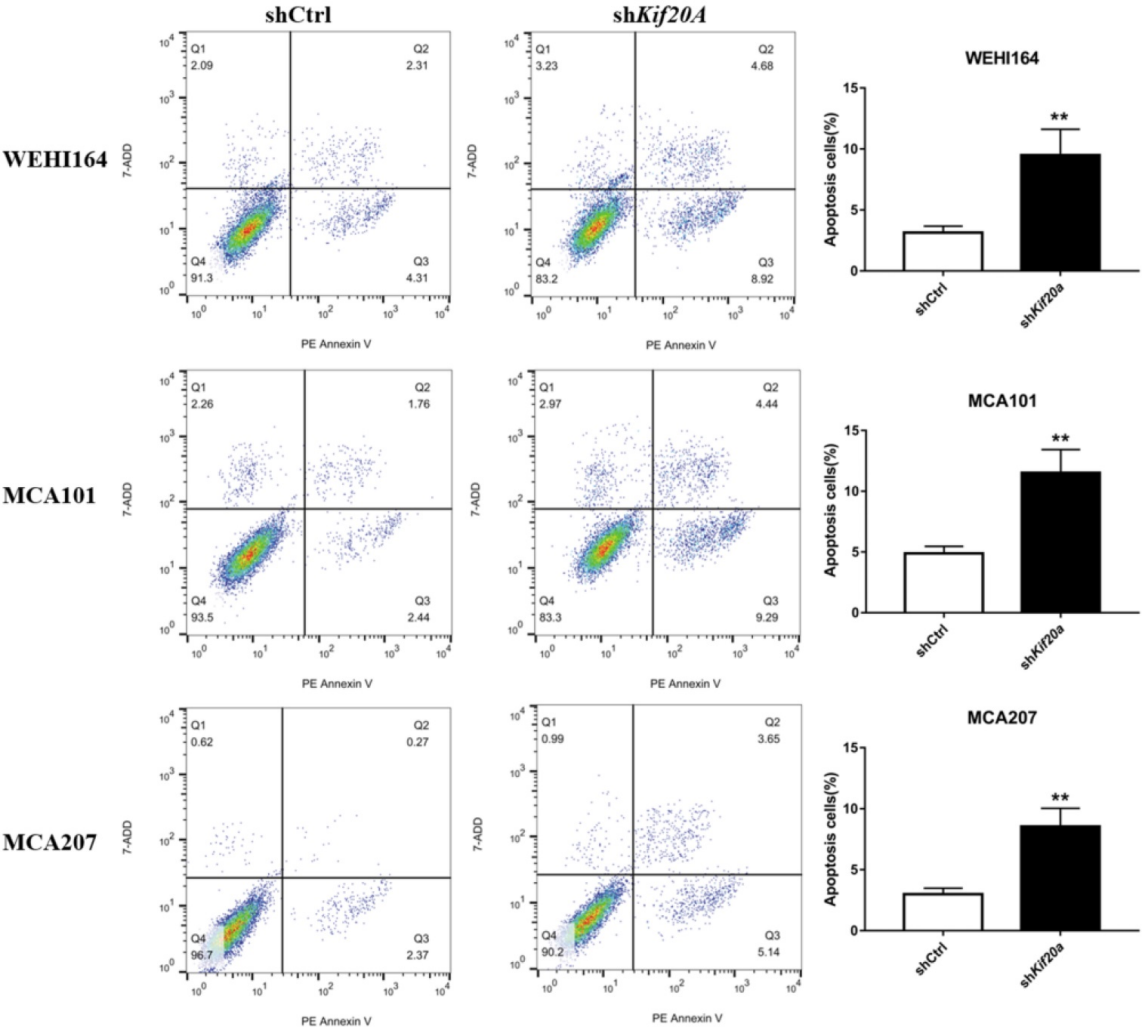
Downregulation of Kif20a promote cell apoptosis *in vitro*. Q2 indicating cells in late apoptosis and necrosis, Q3 indicating cells in early apoptosis.

After *Kif20a* was downregulated, the proportion of cells in the second (late apoptosis and necrosis) and third quadrants (early apoptosis) increased. Apoptosis in WEHI164 cells increased from 6% to 13%, MCA101 from 4% to 13%, and MCA207 from 2% to 8% (Figure 6).

### Downregulation of *Kif20a* inhibits cell migration and cell invasion *in vitro*

**Figure 7:**
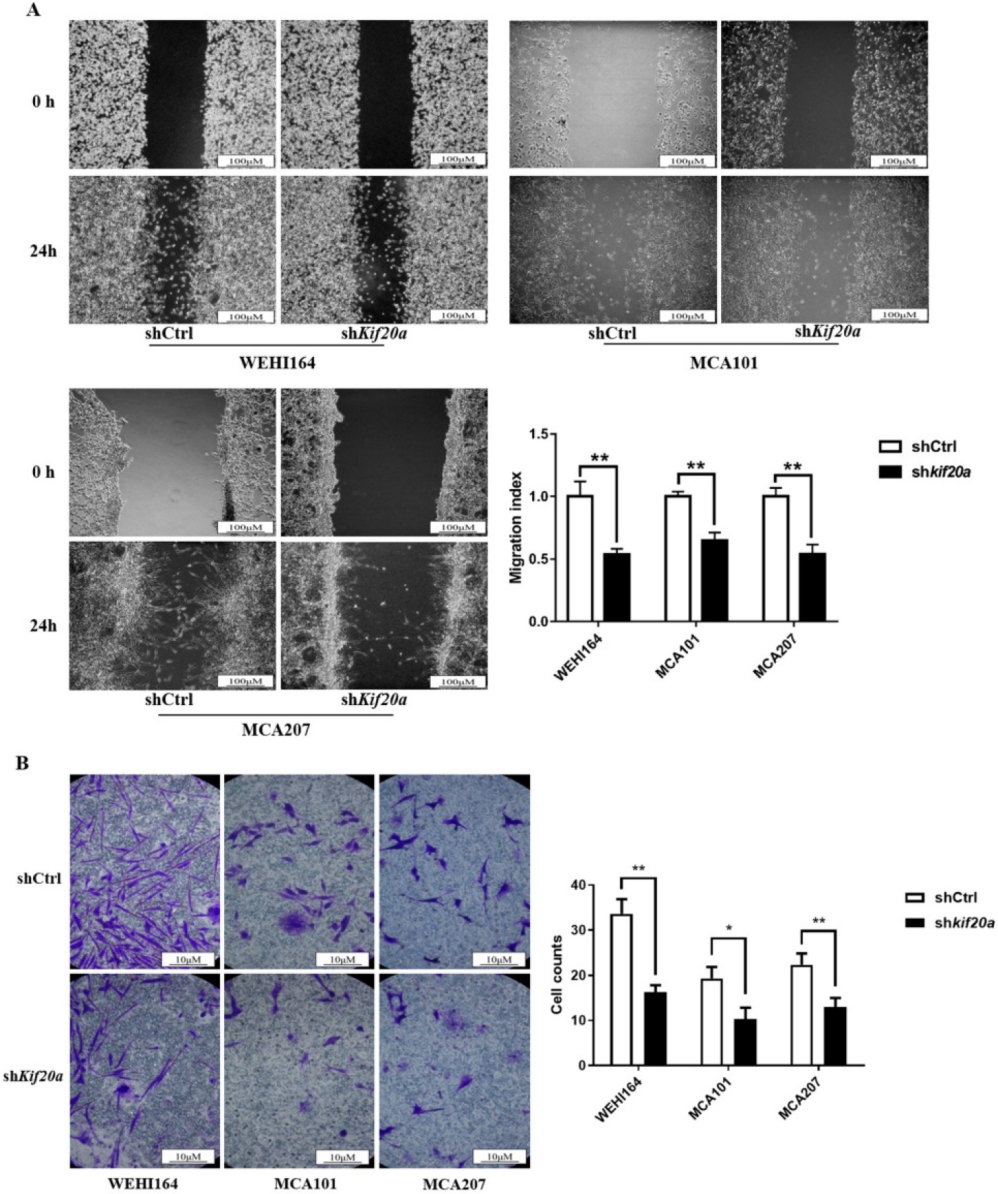
Downregulation of KIF20A inhibited cell migration and cell invasion *in vitro*. (**A**) Cell migration was determined by wound healing assay. (**B**) Cell invasion was determined by transwell invasion assay. *P<0.05, **P<0.01.

To determine the effect of *Kif20a* on the metastasis capability of STS cells, migration (Figure 7A) and invasion (Figure 7B) assays were performed in shCtrl and sh*Kif20a* cells using wound healing and transwell assays. These tests indicated that the capability of the cells in both respects were significantly (*P*<0.05) reduced after Kif20*a* knockdown.

### Downregulation of *Kif20a* suppresses tumor growth *in vivo*

**Figure 8:**
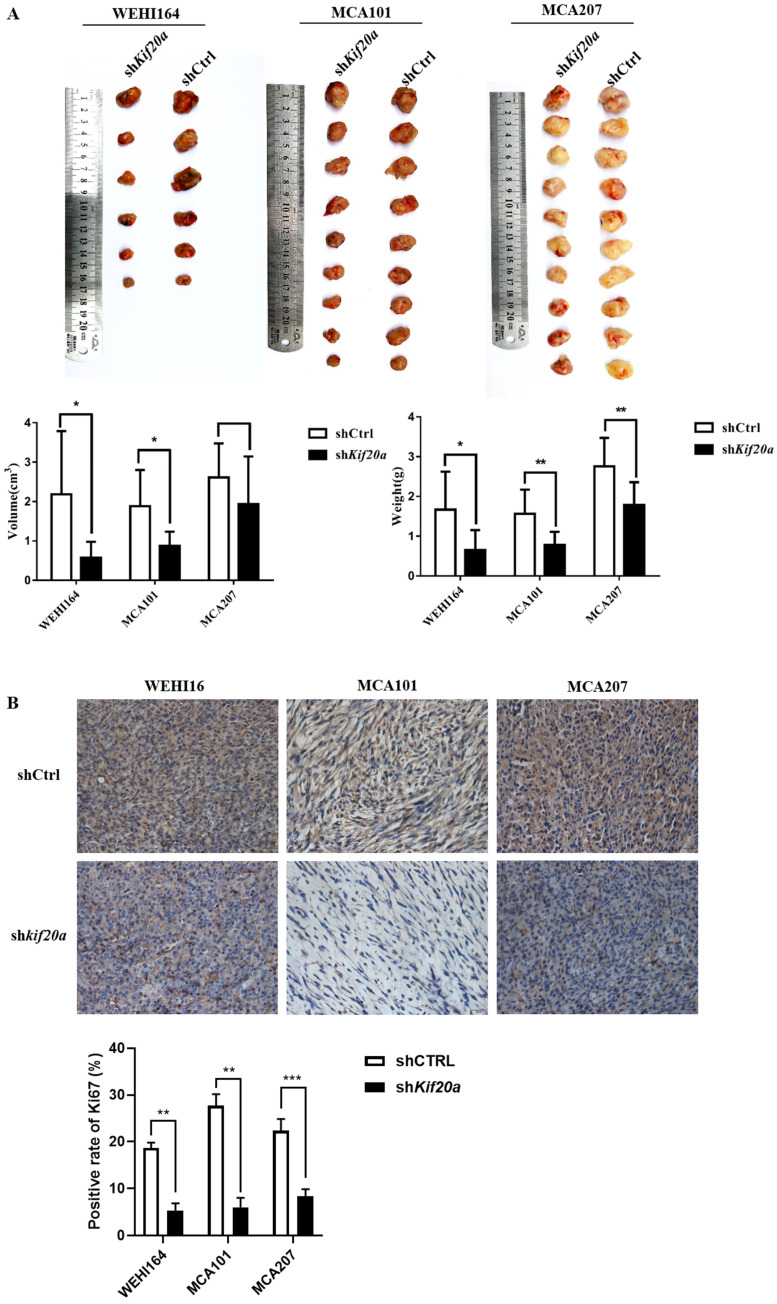
Downregulation of KIF20A suppressed tumor growth *in vivo*. (**A**) Tumor tissues from different treatment groups demonstrating differences in volume and weight of tumors (Left to right: WEHI164, MCA101, MCA207). (**B**) Ki-67 expression in tumors determined by IHC.

To investigate the effect of *Kif20a* on tumor growth, we established a tumor-bearing mouse model. *In vivo* animal experiments demonstrated that tumor growth was slower after knock down (Figure 8A). Analysis of Ki67 by IHC indicated a reduction in tumor proliferation (Figure 8B). We further measured metastasis of the tumor and found no metastasis in the heart, liver, spleen, lung, kidney or brain (Fig. S2). These results reveal that downregulation of Kif20*a* suppressed tumor growth, but not tumor metastasis, *in vivo*.

## Discussion

In this study, we found that high expression of Kif20*a* indicated poor prognosis in STSs. Further analysis indicated that Kif20*a* is related to cell cycle and the p53 signaling pathway. We also confirmed that Kif20*a* is upregulated in STS cell lines. Downregulation of Kif20*a* inhibited proliferation and colony formation *in vitro* and suppressed tumor growth *in vivo*.

STS has a low incidence but poor prognosis. The capillary morphogenesis gene 2 (CMG2) has been demonstrated to promote endothelial cell proliferation and exhibits angiogenic properties. In STSs, low CMG2 expression was correlated with a worsened prognosis 18. In this study, we found that high Kif20*a* expression predicted poor prognosis, which is a new finding.

Kif20*a* is a member of the kinesin superfamily of proteins. It consists of three domains: an N-terminal motor domain, a coiled coil domain and a C-terminal tail domain. The N-terminal domain mediates motor activity, the coiled coil domain interacts with partner proteins and participates in dimerization. The C-terminal tail domain can also interact with partner proteins and contributes to vesicle transport 19. The present study found that Kif20*a* is isolated in the nucleus during G2 phase, thus preventing the cross-linking and depolymerization of cytoplasmic microtubules 20. After the knock-out of Kif20*a*, it was shown that STS cells became stuck in the G2 phase in this study. In tumors, Kif20*a* expression increased significantly, it promoted the growth of tumors and tumor cell lines, and was related to carcinogenesis and aggressiveness 19. The present study demonstrated that Kif20*a* was upregulated in STSs, promoted the growth of STSs, similar to most tumors. In pancreatic cells, Kif20*a* was shown to promote cell motility and invasiveness by transport of RNA-binding proteins 21. We found that Kif20*a* regulated cell division and contributed to tumor growth both *in vitro* and *in vivo* and in STSs. In order to explore the mechanism further, PPI network analysis was conducted, which predicted that Kif20*a* co-expressed with PLK1, INCENP and AURKB. PLK1 is a serine/threonine-protein-like kinase. It can form a complex with Kif20*a* which it phosphorylates, regulating its activity 22. INCENP, AURKB, Survivin and Borealin contain chromosomal passenger complexes (CPCs), which play an important role in regulating the accuracy of the mitotic process. Previous studies have revealed that Kif20*a* participates in the recruitment of the CPC to the spindle 23. We hypothesize that Kif20*a,* along with PLK1, INCENP and AURKB regulate STS, but further evidence is required to confirm this speculation.

It should be noted that due to the lack of normal control samples and sufficient cell lines in this study, the level of Kif20a expression in STSs needs to be further confirmed.

In summary, our findings revealed that Kif20a performs an important role in STS, indicating that it is a potential prognostic biomarker and potentially representing a therapeutic target for the disease.

## Supplementary Material

Supplementary figures.

## Figures and Tables

**Table 1:** Clinical information of TCGA-SARC project

Characteristic	Detail
Age	61(20-90)
**Gender**	
Female	141
Male	118
**Race**	
White	226
American	18
Black or African American	18
Asian	6
Not Evaluated	6
Unknown	3
**Histological type**	
Leiomyosarcoma (LMS)	105
Dedifferentiated liposarcoma	59
Undifferentiated Pleomorphic Sarcoma (UPS)	50
Myxofibrosarcoma	25
Others	22
**Necrosis**	
0% (no necrosis or no mention of necrosis)	70
<10% (focal necrosis)	38
Moderate Necrosis (≥10, <50%)	61
Extensive Necrosis (>50%)	12
Not Available	78
**Radiation therapy**	
NO	140
YES	73
Unknown	10
Not Available	36
**Vital status**	
Alive	184
Dead	75

**Table 2:** The most enriched KEGG pathways of the high KIF20A expression group

NAME	SIZE	NOM p-value	FDR q-value
KEGG_OOCYTE_MEIOSIS	46	0	0
KEGG_CELL_CYCLE	57	0	0
KEGG_P53_SIGNALING_PATHWAY	42	0.001912	7.40E-04
KEGG_PROGESTERONE_MEDIATED_OOCYTE_MATURATION	39	0.001969	0.003036
KEGG_PANCREATIC_CANCER	35	0.00789	0.059943
KEGG_SMALL_CELL_LUNG_CANCER	49	0.018145	0.147014
KEGG_UBIQUITIN_MEDIATED_PROTEOLYSIS	23	0.02554	0.152741
KEGG_PROSTATE_CANCER	41	0.051793	0.310258
KEGG_BLADDER_CANCER	28	0.111765	0.39316
KEGG_PYRIMIDINE_METABOLISM	27	0.121032	0.538301
