# Supplementary material for: Knockdown of Kif20a inhibits growth of tumors in soft tissue sarcoma in vitro and in vivo
Source: J Cancer. 2020 Jun 28;11(17):5088–98. doi: 10.7150/jca.44777 (PMC7378921; doi:10.7150/jca.44777)
Supplement: Supplementary file 1 — Supplementary figures. [file jcav11p5088s1.pdf]

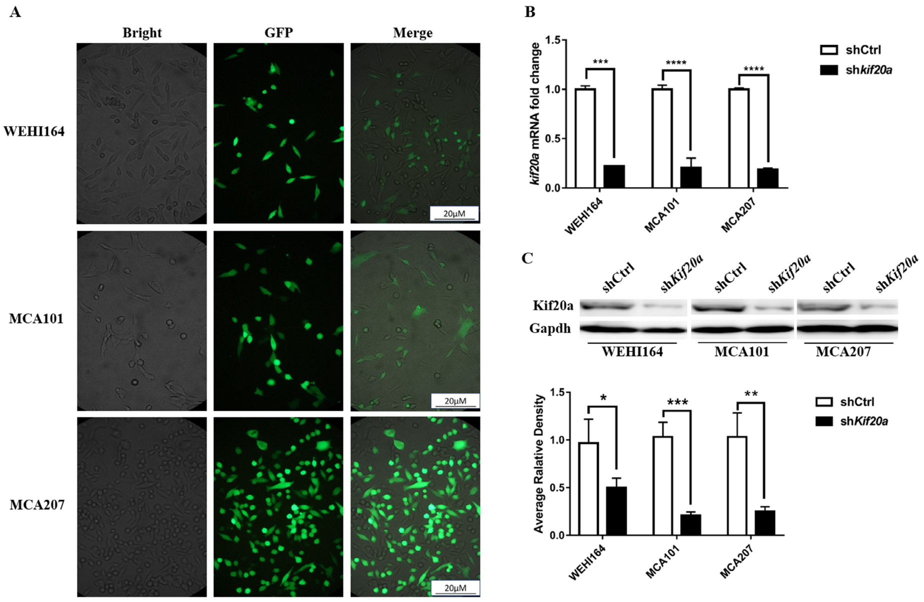


Fig. S1: Knockdown of Kif20a in STS cell lines. (A) Transfection efficiency was determined by fluorescence microscopy, (B) by qRT-PCR (C) and by Western blotting. qRT-PCR and Western blot assays were normalized to GAPDH. *P<0.05, **P<0.01, ***P<0.001, ****P<0.0001.

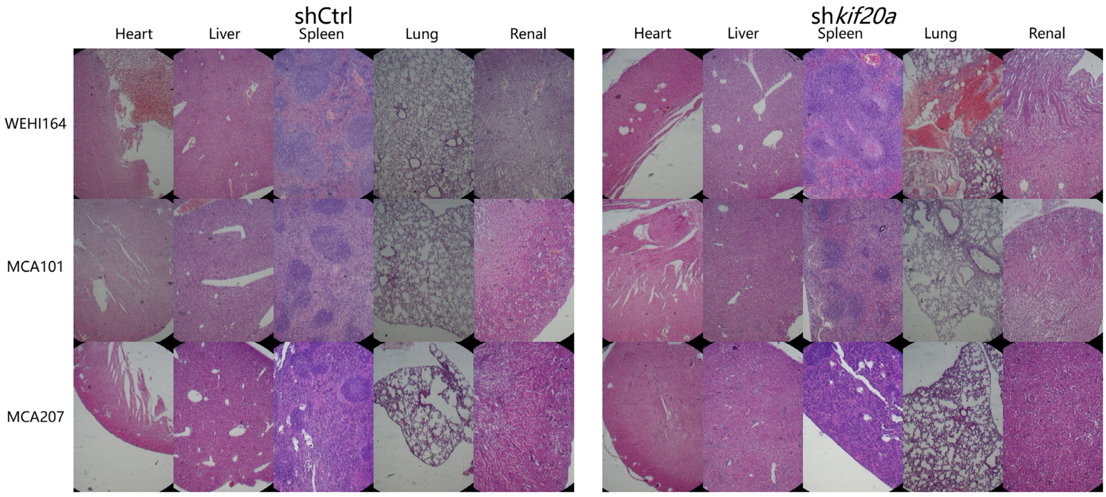


Fig. S2: Tumor metastasis was determined from observation of HE
